# Risk assessment of psychiatric complications in infectious diseases: CALCulation of prognostic indices on example of COVID-19

**DOI:** 10.3389/fpsyt.2024.1341666

**Published:** 2024-02-15

**Authors:** Mikhail Sorokin, Kirill Markin, Artem Trufanov, Mariia Bocharova, Dmitriy Tarumov, Alexander Krasichkov, Yulia Shichkina, Dmitriy Medvedev, Elena Zubova

**Affiliations:** ^1^ Institute of Clinical Psychiatry, V.M.Bekhterev National Medical Research Centre for Psychiatry and Neurology, Saint Petersburg, Russia; ^2^ Psychiatry Department, Kirov Military Medical Academy, Saint Petersburg, Russia; ^3^ Department of Neurology and Manual Medicine of the Faculty of Postgraduate Education, Pavlov First Saint Petersburg State Medical University, Saint–Petersburg, Russia; ^4^ Neurology Department, Kirov Military Medical Academy, Saint Petersburg, Russia; ^5^ Department of Computer Science and Engineering, Saint-Petersburg Electrotechnical University “LETI”, Saint–Petersburg, Russia; ^6^ Department of Old Age Psychiatry, Institute of Psychiatry, Psychology and Neuroscience (IoPPN), King’s College London, London, United Kingdom; ^7^ Radio Engineering Systems Department, Saint-Petersburg Electrotechnical University “LETI”, Saint–Petersburg, Russia; ^8^ Research Centre “Saint Petersburg Institute of Bioregulation and Gerontology”, Saint Petersburg, Russia; ^9^ Institute of Postgraduate Education, V.M.Bekhterev National Medical Research Centre for Psychiatry and Neurology, Saint Petersburg, Russia

**Keywords:** inflammation, biomarkers COVID-19, SARS-CoV-2, psychopathology, ROC curve

## Abstract

**Introduction:**

Factors such as coronavirus neurotropism, which is associated with a massive increase in pro-inflammatory molecules and neuroglial reactivity, along with experiences of intensive therapy wards, fears of pandemic, and social restrictions, are pointed out to contribute to the occurrence of neuropsychiatric conditions.

**Aim:**

The aim of this study is to evaluate the role of COVID-19 inflammation-related indices as potential markers predicting psychiatric complications in COVID-19.

**Methods:**

A total of 177 individuals were examined, with 117 patients from a temporary infectious disease ward hospitalized due to COVID-19 forming the experimental group and 60 patients from the outpatient department showing signs of acute respiratory viral infection comprising the validation group. The PLR index (platelet-to-lymphocyte ratio) and the CALC index (comorbidity + age + lymphocyte + C-reactive protein) were calculated. Present State Examination 10, Hospital Anxiety and Depression Scale, and Montreal Cognitive Assessment were used to assess psychopathology in the sample. Regression and Receiver operating characteristic (ROC) analysis, establishment of cutoff values for the COVID-19 prognosis indices, contingency tables, and comparison of means were used.

**Results:**

The presence of multiple concurrent groups of psychopathological symptoms in the experimental group was associated (R² = 0.28, F = 5.63, p < 0.001) with a decrease in the PLR index and a simultaneous increase in CALC. The Area Under Curve (AUC) for the cutoff value of PLR was 0.384 (unsatisfactory). For CALC, the cutoff value associated with an increased risk of more psychopathological domains was seven points (sensitivity = 79.0%, specificity = 69.4%, AUC = 0.719). Those with CALC > 7 were more likely to have disturbances in orientation (χ² = 13.6; p < 0.001), thinking (χ² = 7.07; p = 0.008), planning ability (χ² = 3.91; p = 0.048). In the validation group, an association (R²_McF_ = 0.0775; p = 0.041) between CALC values exceeding seven points and the concurrent presence of pronounced anxiety, depression, and cognitive impairments was demonstrated (OR = 1.52; p = 0.038; AUC = 0.66).

**Discussion:**

In patients with COVID-19, the CALC index may be used for the risk assessment of primary developed mental disturbances in the context of the underlying disease with a diagnostic threshold of seven points.

## Introduction

The end of the coronavirus pandemic in May 2023 left many issues that could improve the health system as a whole and prepare it to face possible global challenges in the future. One such organizational issue was the provision of specialized care for patients with COVID-19 in the presence or development of comorbid pathology, particularly psychiatric (1). The conditions of the pandemic, which required the conversion of psychiatric wards into infectious disease hospitals and the reduction of face-to-face consultations, provoking a shortage of specialists in specific medical profiles, made it difficult to maintain the mental health of the population (2). Conversely, due to the difficulty of assessing mental pathology in patients with an actual infectious disease by general practitioners or infectious disease specialists, care for this population group in temporary infectious disease hospitals and outpatient services was not always provided promptly (3). At the same time, the imposition of anti-epidemic restrictions in society, fear of the pandemic, and fear of being in intensive care wards were available actual factors that contributed to the development of symptoms of mental disorders: anxiety, depression, and dyssomnia (4).

On the other hand, the role of inflammation in the development and course of mental disorders has been considered quite extensively before (5, 6). For example, in a large study conducted since 2003, it was shown that the elevated levels of C-reactive protein are associated with an increased risk of psychological distress and depression in the general population (7, 8), as well as more, specifically, the role of C-reactive protein levels has recently been discussed in the development of psychiatric complications in COVID-19 (9, 10). However, the detected neurotropism of SARS-COV-2 has raised the question of whether there is a direct link between the severity of the course of the infectious process and mental state, as well as their various bidirectional associations (4, 11, 12). A retrospective analysis of data from 62,000 cases of novel coronavirus infection revealed an increased risk of developing symptoms of psychiatric disorders, primarily anxiety, sleep disturbances, and cognitive decline in patients with advanced novel coronavirus infection, and that having a history of psychiatric diagnosis may be an independent risk factor for COVID-19 (13). In a study evaluating the association of pre-pandemic CRP levels with the likelihood of developing depressive symptoms during a pandemic, it was found that respondents with higher baseline CRP concentrations were 40% more likely to develop depressive symptoms during the pandemic (14).

However, isolated inflammatory markers cannot always predict the presence of comorbid psychiatric symptomatology (15). The use of inflammatory indices in clinical practice is effective in assessing the severity of a patient’s current condition and is predictive of possible deterioration in patients with COVID-19 (16–18). Their significant advantage, in particular, is the composition of the monitored parameters, which are not always as informative as in combination. Moreover, most of the available inflammation indices are calculated on the basis of routine laboratory and instrumental diagnostic methods, which allows them to be used almost universally (19).

### Objective

The aim of this study is to evaluate the role of COVID-19 inflammation-related indices as potential markers predicting psychiatric complications in COVID-19.

## Method

### Materials

A total of 177 individuals were examined: 89 men (50.3%) and 88 women (49.7%). Mean age 50.9 (18.3) years [M (SD)]. One hundred seventeen inpatients from a temporary infectious disease ward for COVID-19 were included in the experimental group (66.1%) after signing their informed consent. For most of them, the main reason for hospitalization was the unfavorable course of COVID-19. Most of them also did not have any previous mental disorders (92.7% of the experimental group).

The inclusion criteria for the experimental group were as follows: 1) ability to read and understand and readiness to sign a voluntary informed consent to take part in the study; 2) a hospitalization due to COVID-19 diagnosis in a temporary infectious disease ward; and 3) ability to fulfill the study procedures (diagnostic interview). The non-inclusion criteria were as follows: 1) extremely high severity of the current condition with insufficient respiratory function and 2) age less than 18 years. The exclusion criteria were as follows: 1) absence of sufficient for the current study clinical data in medical documentation (somatic comorbidity, lymphocyte count, C-reactive protein, etc.) and 2) refusal to comply with the study procedures at any stage of the study. In this portion of the sample, the utilization of systemic indices of inflammation was tested. We evaluated the non-specific platelet-to-lymphocyte ratio (PLR) as a possible predictive index for the risk assessment of psychiatric complications development along the course of COVID-19. The rationale for its use was based on previously obtained data on the role of platelet count in the formation of various psychopathological conditions in patients with COVID-19 (20). In addition, a specific SARS-CoV-2 CALL index was evaluated in slightly modified version to settle its prognostic potential as a tool for risk assessment of psychiatric complications during COVID-19. In contrast with originally developed CALL index (19), we used comorbidity of neurological, endocrinological, or cardiovascular disorders, the age of patients, lymphocyte count, and C-reactive protein instead of lactate dehydrogenase level. Thus, the second inflammation associated index was CALC (comorbidity, age, lymphocyte, and C-reactive protein).

Sixty patients from the outpatient departments with signs of acute respiratory viral infection comprised the validation group (33.9%). Most of them were randomly invited to participate in the current study during an appointment with a general practitioner for outpatient treatment of an acute respiratory viral infection. Most of them also did not have any previous mental disorders (92.9% of the validation group). The inclusion, non-inclusion, and exclusion criteria for validation group were the same as that for the experimental group except the inclusion criteria # 2: outpatients of the North-West region of Russia with the presence of an acute respiratory viral infection. Forty patients in the validation group had PCR-smear or X-ray (CT-based) confirmed diagnosis of COVID-19 (66.7% of the validation group). Clinical and demographic characteristics of the experimental group and the validation group are presented in **Table 1**. It describes the more complicated state of inpatients in terms of demographic risk-factors (age) and general health (somatic comorbidity and C-reactive protein).

**Table 1:** Clinical and demographic characteristics of the sample.

Characteristic	Group	N	Mean (SD)	Median [IQR]	P-level
Age	Experimental	117	56.9 (17.8)	57.0 [27.0]	<0.001
	Validation	60	39.2 (13.1)	39.0 [19.0]	
Males/Females	Experimental	54/63			0.125
	Validation	35/25			
Somatic comorbidity, Yes/No	Experimental	31/86			<0.001
	Validation	33/27			
Psychiatric anamnesis, Yes/No	Experimental	8/109			0.966
	Validation	4/56			
Anxiety disorder/Depressive disorder/Schizophrenia spectrum	Experimental	3/1/4			0.165
	Validation	2/2/0			
С-reactive protein	Experimental	117	31.4 (55.0)	10.0 [29.7]	0.005
	Validation	60	18.1 (32.7)	6.0 [10.0]	
Respiratory rate (per min)	Experimental	117	19.3 (3.3)	19.0 [4.0]	0.262
	Validation	48	18.9 (3.5)	18.0 [4.3]	
Saturation (%)	Experimental	116	95.1 (3.6)	96.0 [4.0]	0.271
	Validation	48	92.2 (10.0)	97.0 [5.0]	
Lung lesion (CT scan stage 0/1/2/3/4)	Experimental	8/49/36/11/2			0.424
	Validation	0/9/4/0/0			

### Assessments

In the experimental group, the inpatients mental state was assessed by 14 resident psychiatrists, neurologists, and psychotherapists using a semi-standardized diagnostic questionnaire, based on “Present State Examination 10” (PSE-10) with binary response options (“yes” or “no”) for specific psychopathological domains. The choice of the clinical semi-standardized interview as a psychopathological assessment tool rather than a psychometric assessment using questionnaires in experimental group was based on the high expected levels of insight decline and formal cognitive impairment in patients with COVID-19 (20, 21). PSE-10 implies that the decision about the presence or absence of a symptom is made by the physician, not the patient. This instrument assumes descriptive psychopathology as a core skill of a specialist in clinical psychiatry also (22). Inpatients in the experimental group were considered to have significant mental disorders if they exhibited disturbances in three or more psychopathological domains.

In the validation group, the prevalence and severity of anxiety and depressive symptoms were assessed using the validated version of Hospital Anxiety and Depression Scale (HADS) (23), and cognitive impairments were assessed using the Montreal Cognitive Assessment (MoCA). Outpatients in the validation group were considered to have significant mental disturbances if they scored seven or more points on the depression and anxiety sub-scales of HADS and less than 25 points on MoCA. For the uniformity of discrete psychopathological characteristics in the experimental and the validation groups, PSE-10 items 2, 3a, and 3b were taken into account to register the presence of anxiety; PSE-10 item 5 to register depressive symptoms; and PSE-10 items 6, 10a, and 13 to register cognitive disturbances in experimental group. For both groups, we calculated the patients’ PLR and the CALC index (comorbidity + age + lymphocyte + C-reactive protein). The latter one was developed on the basis of the criteria of the previously invented CALL index (19) with the lactate dehydrogenase level replaced by the C-reactive protein level.

### Statistical analysis

Statistical software packages (jamovi) were used for linear regression analysis to test the overall predictive potential of PLR and CALC indices in risk assessment of psychiatric complications in COVID-19. ROC analysis in jamovi and an additional package (ppda) was employed to establish cutoff values for the COVID-19 prognosis indices. Contingency table construction and comparison of mean trends were performed to confirm the clinical significance of the established cutoffs. Finally, binomial logistic regression was used to confirm predictive power of inflammation indices with cutoffs in the independent (validation) group of patients.

The study design was controlled by an Independent Ethics Committee, and compliance with the Helsinki Declaration was confirmed in two ethical opinions: in 2020 (EK-I-132-20) and revised in 2023 (EK-I-44/23).

## Results

In the experimental group, 39 patients (33.3% of the group) had three or more domains of psychopathological symptoms in their current mental state. The median number of psychopathological domains (Me [IQR]) was 1[2]. The presence of multiple concurrent groups of psychopathological symptoms in the current mental state of patients in the experimental group, based on linear regression analysis (R² = 0.28, F = 5.63, p < 0.001), was associated with a decrease in the PLR index (OR = −0.004; 95% CI, −0.007 to −0.001; p = 0.004) and a simultaneous increase in CALC (OR = 0.21; 95% CI, 0.08–0.34; p = 0.002). Lung tissue involvement, as indicated by X-ray (CT) scans, also showed associations with more groups of psychopathological symptoms: CT second stage of lung tissue involvement (OR = 1.99; 95% CI, 0.60–3.37; p = 0.005) and CT fourth stage of lung tissue involvement (OR = 2.27; 95% CI, 0.59–5.95; p = 0.017).

An increased risk of a greater number of psychopathological domains was associated with the cutoff value of 70 points for PLR. According to ROC analysis, an AUC for this model was 0.384, which is considered unsatisfactory. For CALC, the cutoff value associated with an increased risk of more psychopathological domains in current mental state was seven points, and a good AUC = 0.719 was revealed. **Table 2** provides evidence supporting the choice of cutoff scores to simultaneously maximize values of sensitivity and specificity as well as the Youden’s index. Cases with PLR indices above 70 points (according to the identified cutoff) were not matched with cases with CALC indices above seven points [χ² = 0.84, d(f) = 1, p = 0.77]. Thus, both of the indices characterized clinically different cases with the more preferable predictive power of CALC (based on AUC calculation). The latter was further assessed more comprehensively.

**Table 2:** Cutoff scores for inflammation-related measures at risk of presenting symptoms in three or more domains of psychopathology.

Cut point	Sensitivity (%)	Specificity (%)	Youden’s index	AUC	Metric score
CALC index (comorbidity + age + lymphocyte + C-reactive protein)					
5	84.21%	39.8%	0.2401	0.719	1.240
6	78.95%	52.04%	0.3099	0.719	1.310
7	78.95%	69.39%	0.4834	0.719	1.483
8	63.16%	79.59%	0.4275	0.719	1.427
9	57.89%	80.61%	0.3851	0.719	1.385
PLR index (platelet-to-lymphocyte ratio)					
63.3	77.78%	18.82%	−0.0340	0.384	0.966
68.0	77.78%	20%	−0.0222	0.384	0.978
70.2	77.78%	21.18%	−0.0105	0.384	0.990
73.6	72.22%	21.18%	−0.0660	0.384	0.934
75.0	72.22%	22.35%	−0.0542	0.384	0.946

Inpatients with CALC values exceeding seven points were older (r_rb_ = 0.675, p < 0.001) and then those with CALC < 7 had higher CT stage of lung lesion (r_rb_ = 0.193, p = 0.026), higher level of C-reactive protein (r_rb_ = 0.585, p < 0.001), and higher respiratory rate (r_rb_ = 0.397, p < 0.001). According to the PSE-10, they had the more simultaneously presented domains of psychopathology in the current mental state (r_rb_ = 0.204, p = 0.008) and were more likely to have disturbances in orientation [χ² = 13.6; d(f) = 1, p < 0.001], thinking [χ² = 7.07; d(f) = 1, p = 0.008], and constructive future planning ability [χ² = 3.91; d(f) = 1, p = 0.048]. It is important to note that patients with a CALC index greater than seven points were no more likely to have previously been diagnosed with mental disorders than those with a CALC < 7 [χ² = 6.07; d(f) = 3, p = 0.108]. In this sense, CALC was more related to current psychopathological symptoms than to previously presented mental illnesses.

**Figure 1:**
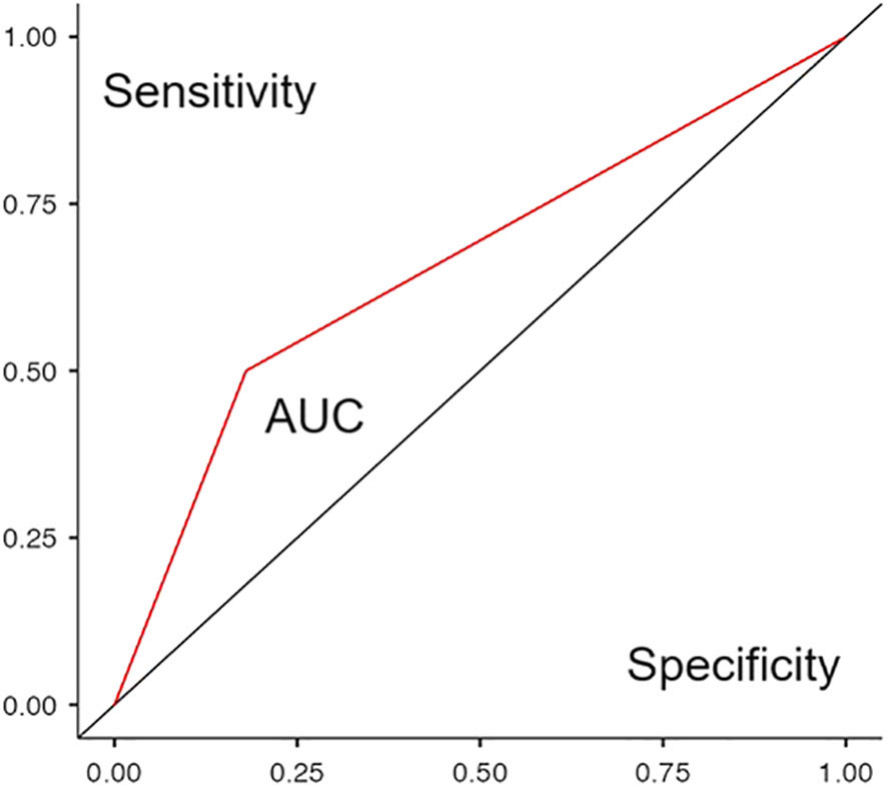
ROC curve of a binomial logistic regression model predicting the presence of significant mental disorders in patients of the validation group based on a dichotomous CALC value of less than or more than seven points.

The predictive value of the established cutoff score for CALC was independently tested in the validation group and compared with the implementation of the total CALC score. Here, 26 patients (43.3% of the validation group) had three or more domains of psychopathological symptoms according to the thresholds presented in the literature for HADS and MoCA scales. Binomial logistic regression model with total CALC score as a predictor of the presence of significant mental disturbances was insignificant (R^2^ **_McF_** = 0.0697; p = 0.052). At the same time the other binomial logistic regression model with the dichotomous CALC value less or exceeding seven points demonstrated an association (R²_McF_ **=** 0.0775; p = 0.041) between CALC values exceeding seven points (OR = 1.52; p = 0.038) and the concurrent presence of simultaneous and pronounced anxiety, depression, and cognitive disturbances. The value of AUC = 0.66 was also satisfactory for the model with dichotomous CALC (**Figure 1**).

## Discussion

The present study demonstrated an association between serum/plasma inflammatory markers and the manifestation of psychopathological symptoms in patients undergoing acute viral infection, specifically COVID-19. The newly developed CALC index, an adaptation of the CALL index (19), exhibited good accuracy in discriminating between patients with and without psychopathological disturbances. However, the discriminative ability of another common inflammation-related index, PLR, did not prove satisfactory.

The divergence in these findings could be attributed to the more complex nature of the population’s psychopathological responses to the COVID-19 pandemic. It has been established that pre-existing psychosocial stress correlates with adverse outcomes in COVID-19 cases (24). Consequently, an index that more accurately represents the patient’s proinflammatory status, one that incorporates general health and age factors like the CALC index, is likely to demonstrate greater accuracy. In the present study, this was evidenced in both the experimental and validation cohorts.

The impact of patient demographics and historical health data on the emergence of psychopathology may be examined through the lens of a network approach to understanding psychopathology (25). This perspective posits that the phenomenological evaluation of mental disorders might rest on the systematic interplay among cognition, emotional states, and behaviors (26, 27). Previous research has also suggested the role of affective state in the presence of mild cognitive impairment in patients with COVID-19 (21). Consequently, individuals perceiving themselves as susceptible to SARS-CoV-2 infection—a notion amplified by media particularly concerning the elderly and those with pre-existing health issues—may have encountered the meditative role of psychosocial stress (28), which, along with the development of coronavirus-related inflammation, may have contributed to the higher incidence of psychopathology in the temporary infection ward.

The recent decade has witnessed a surge in studies of inflammatory biomarkers associated with psychiatric disorders. This growing body of evidence has undeniably enhanced our understanding of the mechanisms underlying mental disorders: inflammation is now known to be intertwined with glucocorticoid neurotoxicity, microglia activation, and neurogenesis (29, 30; https://www.biologicalpsychiatryjournal.com/article/S0006-3223(22)01715-2/fulltext#:~:text=Indeed%2C%20biomarkers%20of%20inflammation%20such,responses%20and%20poor%20clinical%20outcomes); however, the clinical utility of inflammatory markers is yet to be established.

This study represents an example of integrating our basic knowledge (e.g., from multiple studies of CRP associations with psychiatric conditions) with clinical data to develop a prognostic index. The CALC index has shown good discriminative properties in cross-sectional data; future studies should aim to employ longitudinal designs to ascertain the ability of inflammatory markers and indices, specifically CALC, to identify individuals at risk of psychiatric complications. The study’s main strength lies in its naturalistic, non-interventional nature and two-stage design, where the experimental group’s results were confirmed in an independent sample of patients with a similar condition. Another benefit was the ability to set a threshold for the newly developed CALC index to assess better the risk of developing psychiatric complications in patients with COVID-19.

The main limitation of the study was the incomplete number of pathophysiological and psychosocial factors assessed in the sample as possible predictors of psychopathological reactions during SARS-CoV-2 infection. This weakness, in turn, was because the sample size of inpatients who may undergo psychopathological examination in a life-threatening condition was provisionally considered to be limited. Another weakness is the assumption of a causal relationship between neuroinflammation and mental function in patients, which empirically is not always confirmed (10).

## Conclusion

In patients with COVID-19, the prognosis index for the course of the infectious process, CALC (comorbidity + age + lymphocyte + C-reactive protein), may represent a valuable marker for the risk assessment of primary development of mental disorders in the context of the underlying disease. The diagnostic threshold is set at seven points for the prediction of psychiatric complications in inpatients with COVID-19.

## Data availability statement

The raw data supporting the conclusions of this article will be made available by the authors, without undue reservation.

## Ethics statement

The studies involving humans were approved by Independent Ethics Committee of V.M.Bekhterev National Medical Research Centre for Psychiatry and Neurology. The studies were conducted in accordance with the local legislation and institutional requirements. The participants provided their written informed consent to participate in this study.

## Author contributions

MS: Conceptualization, Formal Analysis, Investigation, Methodology, Project administration, Writing – original draft, Writing – review & editing. KM: Conceptualization, Investigation, Project administration, Writing – original draft. AT: Conceptualization, Resources, Writing – review & editing. MB: Formal Analysis, Investigation, Methodology, Writing – review & editing. DT: Resources, Writing – review & editing. AK: Formal Analysis, Resources, Writing – original draft. YS: Formal Analysis, Resources, Writing – review & editing. DM: Resources, Writing – review & editing. EZ: Conceptualization, Methodology, Project administration, Resources, Writing – review & editing.
